# Energy-efficient ultrafast nucleation of single and multiple antiferromagnetic skyrmions using in-plane spin polarized current

**DOI:** 10.1038/s41598-021-91591-8

**Published:** 2021-06-10

**Authors:** Kacho Imtiyaz Ali Khan, Naveen Sisodia, P. K. Muduli

**Affiliations:** Department of Physics, Indian Institute of Technology Delhi, Hauz Khas, New Delhi 110016 India

**Keywords:** Magnetic devices, Spintronics

## Abstract

We numerically investigate the ultrafast nucleation of antiferromagnetic (AFM) skyrmion using in-plane spin-polarized current and present its key advantages over out-of-plane spin-polarized current. We show that the threshold current density required for the creation of AFM skyrmion is almost an order of magnitude lower for the in-plane spin-polarized current. The nucleation time for the AFM skyrmion is found to be $12-7$ ps for the corresponding current density of 1–$3\times 10^{13}~\text{A/m}^{2}$. We also demonstrate ultrafast nucleation of multiple AFM skyrmions that is possible only with in-plane spin polarized current and discuss how the current pulse width can be used to control the number of AFM skyrmions. The results show more than one order of magnitude improvement in energy consumption for ultrafast nucleation of AFM skyrmions using in-plane spin-polarized current, which is promising for applications such as logic gates, racetrack memory, and neuromorphic computing.

## Introduction

Skyrmions are structures having particle-like properties in topologically stable field configurations^1^. In ferromagnets, topologically protected magnetic skyrmions with a definite chirality can be stabilized in the presence of Dzyaloshinskii–Moriya interaction (DMi) and are characterized by an integral topological charge or skyrmion number^2^, $Q = \pm 1$. DMi, an antisymmetric magnetic exchange interaction, can arise in non-centrosymmetric cubic B20-type helimagnets due to the broken inversion symmetry^3–8^. Chiral skyrmions in such systems were experimentally observed first in MnSi^9^ and later in FeGe^10^ near their respective magnetic ordering temperatures. Near the ordering temperature of noncentrosymmetric ferromagnets, a precursor phenomena has been observed due to the coupling of longitudinal and angular order parameter^11^. Recently, the simultaneous existence of both low and high-temperature skyrmions has also been reported in Cu$_2$OSeO$_3$ due to the competition of anisotropic exchange and cubic anisotropy^12–14^. Room temperature skyrmions have also been realized experimentally^15,16^ in thin ferromagnetic films having interfaces of transition metal with heavy metals due to the broken inversion symmetry at the interface, which provides an interfacial DMi^17–22^. Skyrmions with unique physical properties can also be stabilized even in the absence of DMi in frustrated magnets^23^.

The requirement for low threshold current density for the motion of magnetic skyrmions is a key advantage for racetrack memories, skyrmion based microwave oscillators, and logic gate devices offering ultra-high storage density (due to small size) and low power consumption^24–29^. However, the major disadvantage of using ferromagnetic (FM) skyrmions in racetracks is the presence of an additional transverse motion of the skyrmion (along the nanotrack width due to the Magnus force) which eventually leads to the destruction of skyrmion at the nanotrack edge. This phenomenon, called skyrmion Hall effect (SkHE), occurs due to the non-zero topological charge ($Q=\pm 1$) and has been observed in several experiments^30–32^.

On the other hand, antiferromagnetic (AFM) skyrmions could be a better alternative to FM skyrmions for racetrack memory devices due to their trivial topology (zero topological charge) which completely suppresses the skyrmion Hall effect^33,34^. Fractional AFM skyrmion lattice has been recently observed experimentally in spinel MnSc_2_ S_4_ consisting of three antiferromagnetically coupled sub-lattices^35^. The predicted velocity of AFM skyrmions is much higher compared to that of the FM skyrmion^36–40^ and also the oscillation frequency of AFM skyrmion based spin torque nano oscillator (STNO) is much higher as compared to FM skyrmion based STNO^41^. A topological spin Hall effect is also predicted theoretically for the nontrivial magnetic texture of AFM skyrmions^42^. The dynamics of the moments of antiferromagnet falls in the ultrafast (THz regime), which is an additional advantage of these materials in spintronics applications^43–45^. Several methods have been proposed to nucleate AFM skyrmion such as by spin polarized current^36,46,47^, and by use of short laser pulse^48^. So far the studies on nucleation of AFM skyrmions using spin polarized current have focused on out-of-plane spin polarized current, which needs an out-of-plane magnetized spin polarizing layer for operation.

**Figure 1:**
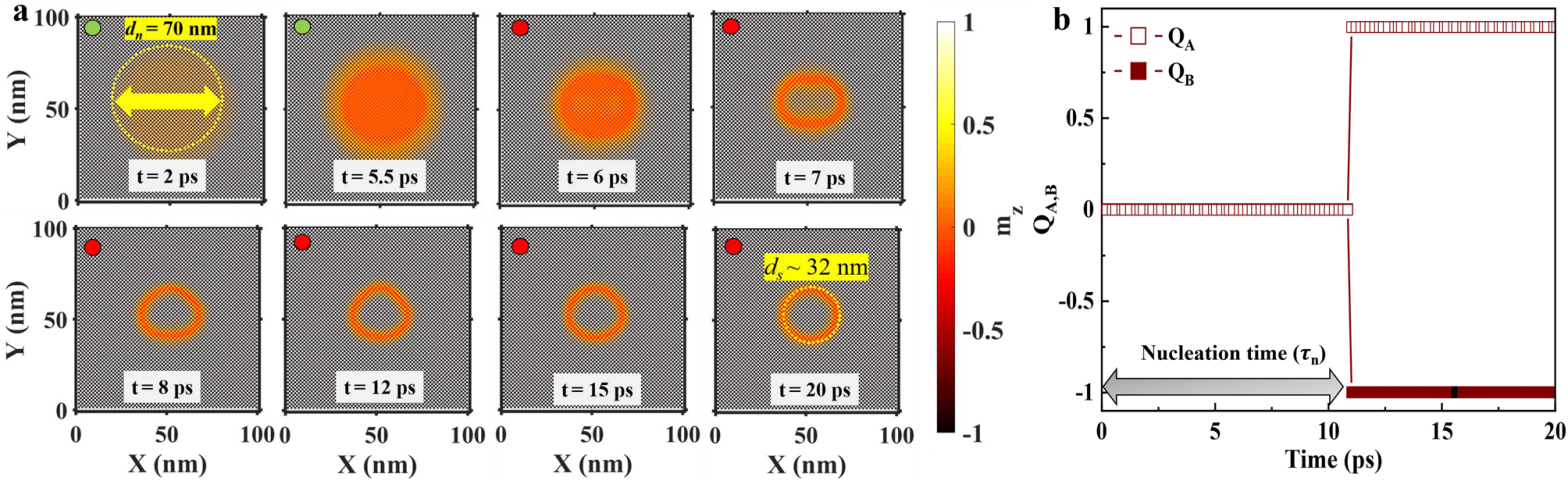
(**a**) Snapshots during nucleation of AFM skyrmion at different instants of time when a current pulse with a width $\tau_{w} =5.5$ ps is applied. Here, (green/red) circle represent current pulses (ON/OFF). The current is injected in a circular region with diameter, d$_n =70$ nm. The final diameter of the AFM skyrmion is found to be d$_s =32$ nm after 20 ps. (**b**) The evolution of topological charge $Q_{\text{A}}$ for sublattice *A* (open symbols) and $Q_{\text{B}}$ of sublattice *B* (closed symbols) during the nucleation of the AFM skyrmion.

In this report, we numerically study the ultrafast nucleation of AFM skyrmion by injecting a spin polarized current density ($J_{\nu ,\mu }$) in an AFM thin film. Here $\nu$ represents its spin polarization direction and $\mu$ represents the direction of spin current. The current is injected in CPP (current perpendicular to plane)^49^ geometry ($\mu ={\hat{z}}$) and the polarization can be both in-plane ($\nu ={\hat{x}}$) or out-of-plane ($\nu ={\hat{z}}$) direction. Our findings show that the threshold current density required for the nucleation of AFM skyrmion using in-plane polarized spin current ($J_{x,z}$) is almost one order less compared to the commonly utilized out-of-plane polarized spin current ($J_{z,z}$). We attribute this difference to the characteristic trajectories of the oscillation eigenmodes excited in the two cases due to the phenomenon of spin transfer torque (STT). We discuss the local switching of magnetic moments under STT for $J_{x,z}$ and $J_{z,z}$ which is required to induce the nucleation of AFM skyrmion. Then, we report on the successive creation of multiple AFM skyrmions which is possible only using in-plane spin polarized current for pulse width, $\tau >\tau _w^0$, where $\tau _w^0$ represents a threshold pulse width for nucleating a single AFM skyrmion. Finally, we check the robustness of our method over a finite range of temperature and we observe that our nucleation method shows a lower threshold pulse width at room temperature.

## Results and discussion

### Micromagnetic simulations

The dynamics of magnetic moments of each sublattice m$_{i}$ (where, $i=A, B$) in AFM are governed by the coupled Landau Lifshitz Gilbert (LLG) equation, with an additional Slonczewski spin torque term originating either from the adjacent heavy metal via spin Hall effect^36,50,51^ or due to the spin transfer torque from a ferromagnetic spin polarizing layer;

1 $$ \begin{aligned} \frac{d {\mathbf {m}}_{i}}{ dt } = - \gamma ({\mathbf {m}}_{i} \times \mathbf {{{}H}_{i}^{eff}}) + \alpha \left( {\mathbf {m}}_{i} \times \frac{d{\mathbf {m}}_{i}}{dt} \right) - \beta \gamma [ {\mathbf {m}}_{i} \times ({\mathbf {m}}_{i}\times \mathbf {\nu})+\xi ({\mathbf {m}}_{i} \times \nu)] \end{aligned}$$

where, $\gamma$ is gyromagnetic ratio of electron, $\alpha$ is the Gilbert’s damping constant and $\beta$ represents the strength of the torque due to spin-current $\left( \beta =\frac{J_{\nu ,\mu } \hbar P}{2 e d M_{s}}\right)$, where $J_{\nu ,\mu }$ represents the amplitude of current density and *P* is the efficiency of spin polarization^52,53^. $M_s$ and *d* are the saturation magnetization and thickness of the AFM layer, respectively. $\nu$ is the polarization direction of electrons and $\xi$ is the ratio of field-like torque to the damping-like torque. H$_{i}^{\text{eff}} = -\frac{1}{\mu _0 M_s} \frac{\delta W_{\text{total}}}{{\delta m}}$ is the effective field, where $W_{\text{total}}$ is the total magnetic energy which contains energy density contributions from exchange interaction ($W_{\text{ex}}$), DMi ($W_{\text{dmi}}$) and magnetic anisotropy ($W_{\text{anis}}$), as given below^54^,

2 $$\begin{aligned} W_{\text{{total}}}= & {} \int W_{\text{{ex}}} + W_{\text{{dmi}}} + W_{\text{{anis}}} \; dV \nonumber \\&{\text{{where,}}}\nonumber \\ W_{\text{{ex}}}= & {} A_{ex}\left[ \left( \frac{\delta {\mathbf {m}}}{\delta x}\right) ^2+\left( \frac{\delta {\mathbf {m}}}{\delta y}\right) ^2\right] ,\nonumber \\ W_{\text{{dmi}}}= & {} -D \left[ m_x\frac{\delta m_z}{\delta x} - m_z\frac{\delta m_x}{\delta x} + m_y\frac{\delta m_z}{\delta y} - m_z\frac{\delta m_y}{\delta y}\right] \nonumber \\ W_{\text{{anis}}}= & {} K (1-m_{z}^{2}) \end{aligned}$$

here, $A_{ex}$, *D* and *K* represent the exchange constant, the DMi constant and the perpendicular uniaxial anisotropy constant, respectively. It is important to note that even though the partial derivatives in Eq. (2) are not valid for the discontinuous magnetization of an AFM, the numerical solution in Mumax$^3$ (Ref. ^55^) is obtained on a finite difference grid. Thus, each of the terms in Eq. (2) can be reduced to their corresponding atomistic expressions for small cell sizes as shown in Ref. ^54^. The parameter values used for AFM are based on previous works by Menezes et al.^54^ and Zhang et al.^56^ (see “Methods”). For the chosen parameters ($D=3.5~ \rm {mJ/m}^2$; $K=0.8\times 10^6~\rm {J/m}^3$), the AFM skyrmion state is a meta-stable state. However, slightly reducing the anisotropy to $K=0.6\times 10^6~\rm J/m^3$ can lead to the elongation of skyrmion, giving a distorted AFM skyrmion, as shown in Ref. ^56^. Increasing the DMI to $D=3.75~\rm {mJ/m}^2$ (for $K=0.8\times 10^6~\rm {J/m}^3$), gives a ground state AFM skyrmion with lower energy than the checkerboard AFM ground state.

To understand the topology of an AFM skyrmion, we consider an AFM skyrmion to be composed of two separate sub-lattices (sub-lattice A and B), with opposite core polarity ($Q_{\text{A}}=+1$ and $Q_{\text{B}}=-1$) so that its net topological charge is zero. We individually calculate the value of the topological charge $Q_s$ in each of the sub-lattices using the lattice based approach^57^:

3 $$\begin{aligned} \begin{aligned} Q_{s}&= \frac{1}{4\pi }\sum _{i,j,k} q^{s}_{ijk}, \quad \text{{where}} \; q^{s}_{ijk} = 2\arctan \left[ {\frac{{\mathbf {m}}_{i}^{s}\cdot ({\mathbf {m}}_{j}^{s} \times {\mathbf {m}}_{k}^{s})}{1+{\mathbf {m}}_{i}^{s}\cdot {\mathbf {m}}_{j}^{s}+{\mathbf {m}}_{i}^{s}\cdot {\mathbf {m}}_{k}^{s}+{\mathbf {m}}_{j}^{s}\cdot {\mathbf {m}}_{k}^{s}}}\right] \end{aligned} \end{aligned}$$

here, index *s* denotes the sub-lattice *A* or *B*. *i*, *j* and *k* are the indices of the unique triangles formed by three nearest neighbour cells. We adopt this approach for computing $Q_s$ since it is shown to be more accurate especially for the finite temperature as compared to the method that uses spatial derivatives of ${\mathbf {m}}$ calculated using the nearest-neighbouring finite difference cells^57^.

### Nucleation of single AFM skyrmion

In Fig. 1a, we nucleate an AFM skyrmion by locally injecting an in-plane spin polarized current ($J_{x,z}$) in CPP configuration. We start with a Néel or checkerboard type AFM configuration which is the ground state of our system with energy $W_0$ and inject (at $t=0$ ps) an in-plane spin polarized current ($\nu = +x$) with current density $J_{{x},{z}}=1 \times 10^{13}~\rm {A/m}^2$ at the centre of the thin film within a circular area of diameter 70 nm as shown in the panel corresponding to $t=2~\rm ps$ in Fig. 1a. Due to the in-plane spin polarization of injected spin current, the magnetic moments in the AFM experience a torque leading to a precessional motion of spins. We switch-off the current after time $\tau _w=5.5~\rm ps$ which we define as the current pulse width.

**Figure 2:**
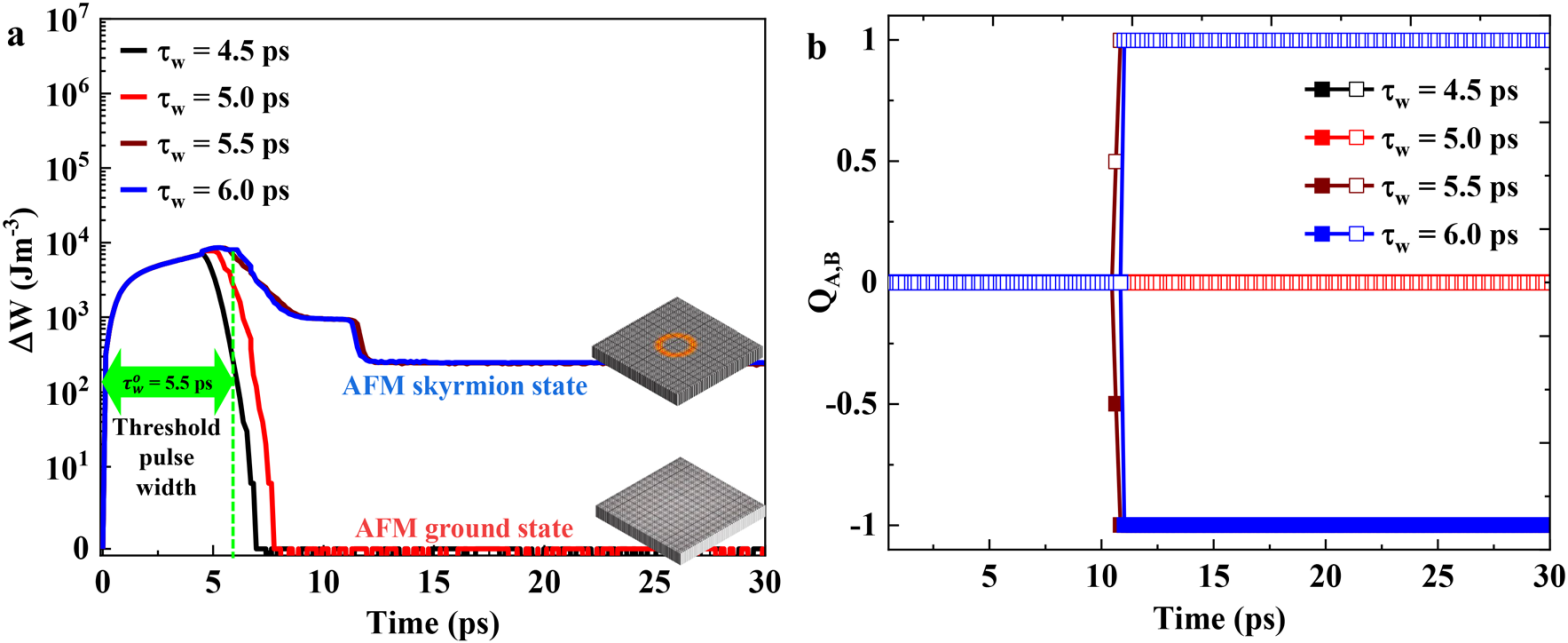
(**a**) Change in total energy density $\Delta W$ during the nucleation of AFM skyrmion for varying pulse widths ($\tau _{w}= 4.5,~5.0,~5.5$, and 6.0 ps). (**b**) The corresponding evolution of topological charge $Q_A$ for sublattice *A* (open symbols) and $Q_B$ of sublattice *B* (closed symbols).

Once the current pulse is switched off ($t>\tau _w$), the system is allowed to gradually relax (energy minimization) to a stable state, which in our case is an AFM skyrmion with 32 nm diameter as shown at $t=20~\rm ps$ in Fig. 1a. A complete video of this nucleation process can be found in the Supplementary Movie 1. To further confirm the formation of AFM skyrmion, we use Eq. (3) to examine the evolution of topological charge $Q_A$ and $Q_B$ of individual sublattice *A* and *B*, respectively (Fig. 1b). It can be clearly seen that both $Q_A$ and $Q_B$ acquire the expected integral value of $\pm 1$ after $t\approx 11$ ps. The opposite sign of topological charge for each sub-lattice also confirms the anti-ferromagnetic nature of the skyrmion topology. We define the total time required for the topological charge $Q_A$ and $Q_B$ to attain a value of $\pm 1$ as the nucleation time ($\tau _n=11$ ps) for the AFM skyrmion.

In Fig. 2a, we show the evolution of the total energy density of the system with reference to the energy density of the initial checkerboard AFM state ($\Delta W=W_{\text{{tot}}}-W_0$) for different values of pulse width, $\tau _w$. For all cases, it was observed that on the current injection, the total energy of the system first increases due to the additional torque arising from the injected spins. Once the current is switched off (t $>\tau _w$), the energy starts to decrease due to the damping part of LLGS equation and eventually saturates at a finite value. It was observed that for cases with $\tau _w<5.5$ ps, the system relaxes back to the initial checkerboard AFM state with $\Delta W=0$ aJ as t$\rightarrow \infty$. For $\tau _{w}\ge 5.5$ ps, the system relaxes to a finite $\Delta W$ value which represents AFM skyrmion state. We define this pulse width as the threshold pulse width ($\tau _{w}^{0}$) to nucleate an AFM skyrmion. Figure 2b shows the value of topological charge for individual sub-lattices for $\tau _{w}<5.5$ ps and $\tau _{w}\ge 5.5$ ps which confirms that for $\tau _w<5.5$ ps, no topological state is achieved in any of the sub-lattices ($Q_{\text{A}}=Q_{\text{B}}=0$) compared to the $\tau _w\ge \tau _{w}^{0}=5.5$ ps case where, in the final state both sub-lattices host a skyrmion with opposite core polarity ($Q_{\text{A}}=-1$, $Q_{\text{B}}=+1$) giving an overall AFM skyrmion state. The obtained AFM skyrmion state is a meta-stable state of the system since the total energy for this state is higher than the energy of checkerboard AFM state ($\Delta W>0$). We also note that for $\tau _w\ge \tau _{w}^{0}=5.5$ ps, although the AFM skyrmion is always nucleated, the number of AFM skyrmions nucleated may be more than one depending on the length of the pulse width. This will be discussed in detail later.

**Figure 3:**
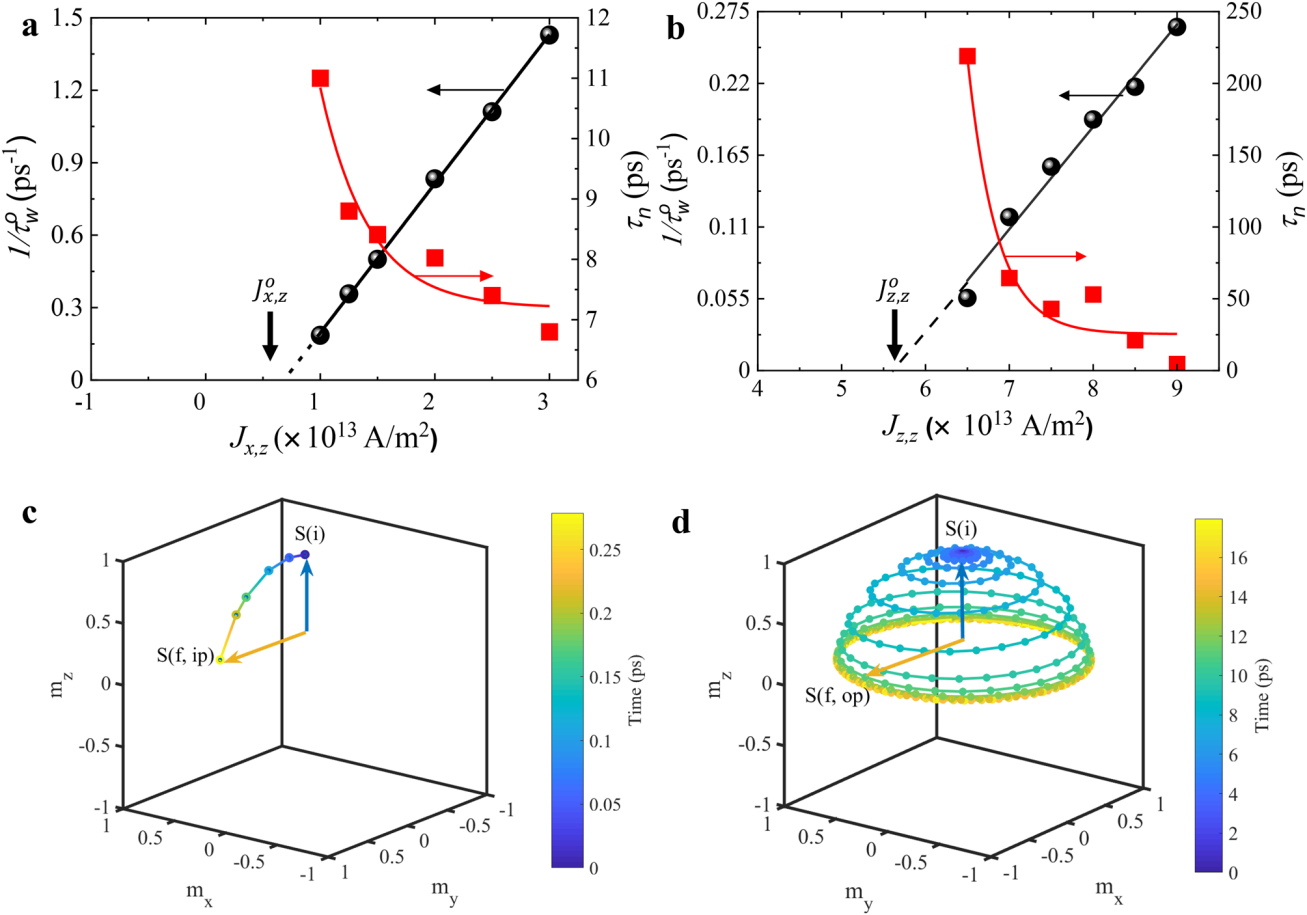
Dependence of inverse of threshold pulse width (black circles) and nucleation time (red squares) on the current density for (**a**) in-plane, and (**b**) out-of-plane spin polarized cases. The trajectory of single AFM moments at fixed current density ($J_{{x},{z}}=J_{{z},{z}}=6.5 \times 10^{13}~\rm {A/m}^2$) for (**c**) in-plane and (**d**) out-of-plane cases. The color in (**c**) and (**d**) represents time elapsed and $t=0$ ps represents the time instant when the pulse is injected.

The threshold pulse width $\tau _w^0=5.5$ ps calculated from Fig. 2 corresponds to a current density of $J_{{x},{z}}=1 \times 10^{13}~\rm {A/m}^2$. With a change in the injected current density, $\tau _w^0$ also varies. This is shown in Fig. 3a, where we plot the inverse of threshold pulse width ($1/\tau _w^0$) as a function of injected current density ($J_{x,z}$). We find that $1/\tau _w^0$ increases linearly with increasing current density, i.e., for a higher injected current density, the threshold pulse width is smaller. We also plot nucleation time ($\tau _n$) as a function of the current density in Fig. 3a. We find that the nucleation time is also lower for higher current density giving rise to ultrafast AFM skyrmion creation in $7-12$ ps time scale for $J_{x,z}=1$–$3\times 10^{13}~\rm {A/m}^2$. Using a linear fit for the variation of $1/\tau _w^0$ with current density, we can extrapolate the value of the lowest current density above which the skyrmion can be nucleated. At this value, the nucleation time for the AFM skyrmion will diverge to infinity. We term this minimum value of current required to nucleate the skyrmion (irrespective of the length of current pulse) as threshold current density represented by $J_{x,z}^0$. From the linear fit in Fig. 3a, we obtain the threshold current density of $J_{x,z}^{0}=(0.68\pm 0.02)\times 10^{13}~{A/m}^2$ for the current spin-polarized in the in-plane direction.

### In-plane versus out-of-plane spin polarization

We now compare the nucleation of AFM skyrmion using in-plane spin polarized current ($J_{x,z}$) discussed above with that of out-of-plane spin polarized current ($J_{z,z}$) as studied in previous works^36,46,56^. We perform an independent set of simulation with out-of-plane polarized current ($\nu =+z$) with the same parameters that we have used for in-plane polarized case. For comparison, we consider two key parameters: (1) energy consumption for the AFM skyrmion nucleation and (2) nucleation time.

Figure 3b, shows the variation of $1/\tau _w^0$ and $\tau _n$ as a function of current density ($J_{z,z}$) for the case of out-of-plane spin polarized current. As noted earlier, the current is flowing through a contact at the center of the film in CPP geometry. Similar to the case of Fig. 3a, both $\tau _w^0$ and $\tau _n$ decrease with increasing current density. However, the threshold current density required for AFM skyrmion nucleation in out-of-plane case is $J_{{z},{z}}^0=(5.65\pm 0.14) \times 10^{13}~\rm {A/m}^2$, which is almost eight times higher than the in-plane case $J_{{x},{z}}^0=(0.68\pm 0.02) \times 10^{13}~\rm {A/m}^2$. In addition, the threshold pulse width required for AFM skyrmion nucleation at current ($J_{{x},{z}}=6.5 \times 10^{13}~\rm {A/m}^2$) just above threshold current density $J_{{z},{z}}^0$ is much higher ($\approx$ 60 times) for the out-of-plane case ($\tau _w^0\sim 18.0$ ps) than the in-plane case ($\tau _w^0\sim 0.3$ ps). As the energy consumption is proportional to $J_{\nu ,\mu }^2 \times \tau _w^0$, it is clear that the nucleation using in-plane spin polarized current is significantly more efficient than out-of-plane spin polarized current as both the current density $J_{\nu ,\mu }$ as well as $\tau _w^0$ are lower for in-plane case (Fig. 3a). The quantity $J_{\nu ,\mu }^2 \times \tau _w^0$ is $12.7\times 10^{14}\text A^{2}\rm {s/m}^4$ for in-plane case while it is as large as $760.5\times 10^{14}\;\text A^{2}\rm {s/m}^4$ for out-of-plane case with same current density of $6.5\times 10^{13}~\rm A/m^2$. Thus, the energy consumption in the case in-plane spin polarized current is more than one order of magnitude lower compared to the out-of-plane case.

The second key parameter to compare is the nucleation time. By our definition, this is the total time required for the nucleation of AFM skyrmion (including the duration for which current pulse is on, *i.e.*, $\tau _w$). For the out-of-plane case in Fig. 3b, the nucleation time is $\tau _n\sim 210$ ps for the current density of $J_{{z},{z}}=6.5 \times 10^{13}~\rm {A/m}^2$. This decreases to a value of $\tau _n\sim 54$ ps with a small increase in current density ($J_{{z},{z}}=7 \times 10^{13}~\rm {A/m}^2$). Increasing the current density to $J_{{z},{z}}=9 \times 10^{13}~\rm {A/m}^2$, the nucleation time is reduced further $\tau _n\sim 10$ ps. This is comparable to the nucleation time for in-plane case in Fig.3a at $J_{{x},{z}}=1.0 \times 10^{13}~\rm {A/m}^2$. Therefore, from the comparison of Fig. 3a,b, it is clear that to achieve similar nucleation times, significantly less current is required in the in-plane case compared to the out-of-plane case. Faster nucleation can only be achieved in the out-of-plane case at the expense of using higher current density which would have a detrimental effect on the energy consumption of the device. To understand the results of Fig. 3a,b, we examine the mechanism of nucleation of AFM skyrmion in both cases.

From our micromagnetic simulations, we have found that in both cases (in-plane and out-of-plane), for the nucleation of AFM skyrmion to be successful, the spins have to be rotated at least till an in-plane configuration is achieved [See Supplementary Movies 2, 3]. If the current is switched off before this state, the magnetization will again re-orient and form the checkerboard AFM state similar to the case of $\tau _w<5.5$ ps in Fig. 2. This is also in agreement with the observed threshold nature of the nucleation process in Fig. 3a,b. For a fixed $\tau _w$, the current has to be strong enough to rotate the magnetization just under the injection region to an in-plane state^58^. Similarly, for a fixed current density $J_{\nu ,\mu }$, the pulse width should be large enough for the magnetization to achieve the in-plane state. However, as discussed earlier, there also exists a minimum threshold current density below which the AFM skyrmion cannot be nucleated irrespective of the value of $\tau _w$. For these values of current, the torque due to STT is not sufficient for the magnetization to rotate significantly. To explain how the rotation of magnetization occurs, we plot a representative trajectory of a single magnetic spin at the center of the injection region for both $J_{x,z}$ and $J_{z,z}$ in Fig. 3c,d, respectively. We use the same current density for both cases ($6.5\times 10^{13}~\rm {A/m}^2$). The initial state is shown by the solid blue arrow pointing up and is denoted by $S_i$ for both cases. The final states are denoted by $S_{f,ip}$ and $S_{f,op}$ for in-plane and out-of-plane polarized currents, respectively. The line joining the initial and final states represents the trajectory of the spin. The color of the trajectory at any point directly corresponds to the time elapsed (assuming current injection starts at t = 0 ps) which is shown by the color bar. Since we are dealing with an AFM, we note that the magnetic spins adjacent to the shown spin will trace an opposite trajectory as they are anti-ferromagnetically coupled. From Fig. 3c,d, we find that for the case of $J_{x,z}$, the plane of rotation of magnetization is *y*–*z* while for the case of $J_{z,z}$ the magnetization is precessing in *x*–*y* plane. The frequency of precession for both cases is $\sim 1$ THz. For $J_{x,z}$, the rotation along the *y*–*z* plane is advantageous as it directly pushes the magnetization towards the in-plane direction. The time required for magnetization to reach in-plane state will be roughly 1/4 of the total period of precession cycle. For the case of $J_{z,z}$, the trajectory is very different as the precession is along *x*–*y* plane. Due to this the magnetization gradually moves to in-plane state over a period of several cycles. This can be clearly seen in Fig. 3d from the color of the trajectory which represents the elapsed time. For $J_{x,z}$, the in-plane state is achieved in sub-picosecond range while for $J_{z,z}$ we require $\sim 18$ ps. This also explains why the nucleation time is higher for out-of-plane spin polarized current compared to in-plane spin polarized current.

**Figure 4:**
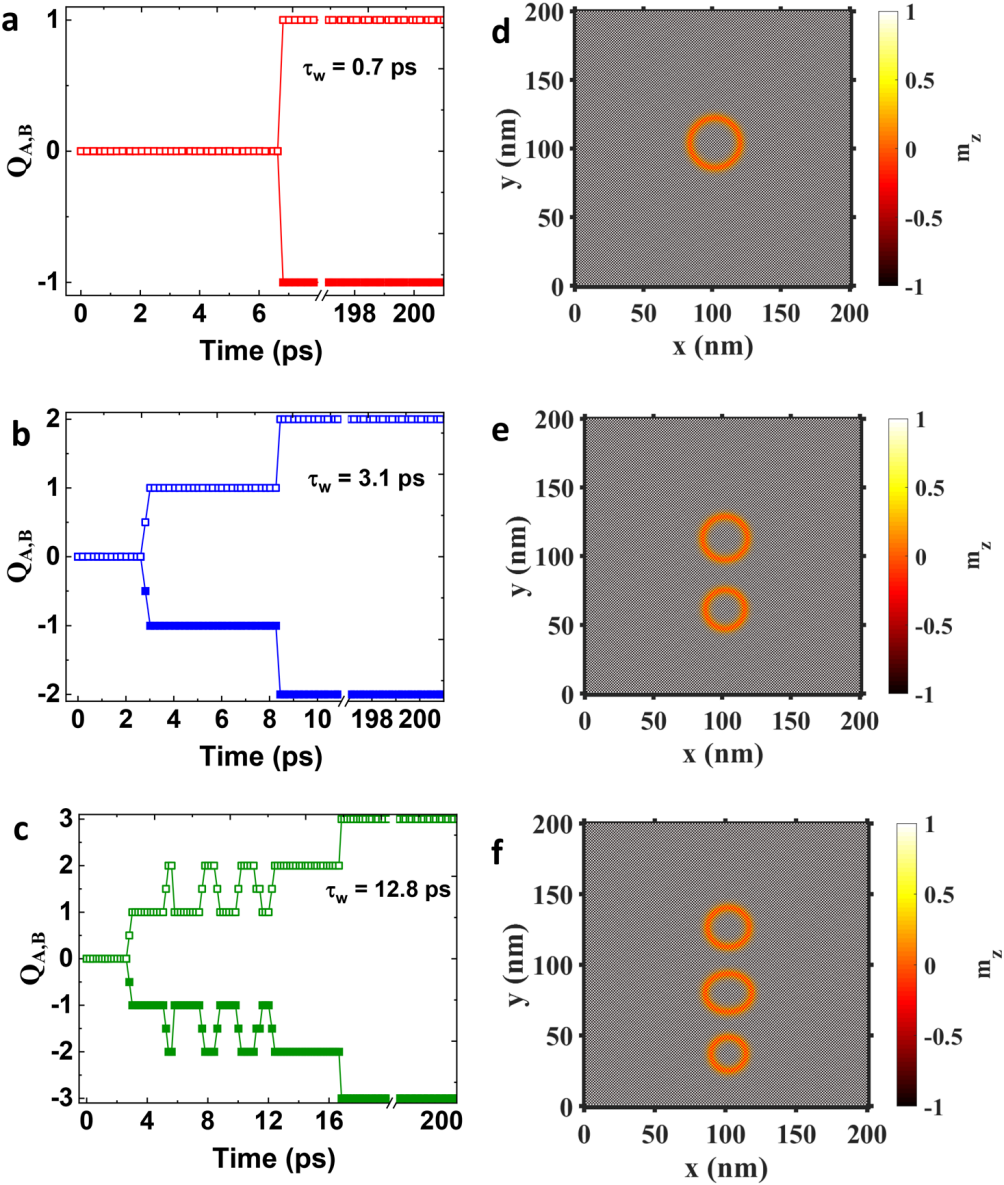
Left figures (**a**–**c**) show the evolution of topological charge for the AFM skyrmion sub-lattices *A* (open symbols) and sub-lattice *B* (closed symbols) for three different pulse widths ($\tau_{w}=0.7, 3.1$ and 12.8 ps) for a constant current density ($J_{x,z}=3 \times 10^{13}$ A/m$^2$) . Right figures (**d**–**f**) show the corresponding snapshots of AFM skyrmions at 200 ps for each case.

### Nucleation of multiple AFM skyrmion

In-plane spin polarized current can also be used for successive nucleation of multiple AFM skyrmions by continuous injection of current for a duration beyond the required threshold pulse width ($\tau _w^0$). We show this property in Fig. 4, where AFM nucleation using in-plane spin polarized current ($J_{x,z}$) is shown for three values of pulse widths ($\tau _w=0.7,~3.1,~12.8~\rm ps$). The current density is kept constant at $J_{x,z} = 3\times 10^{13}$ A/m$^2$. Note that all three values of pulse widths used are above the threshold pulse width for this current density ($\tau _w^0=0.7$ ps).

It can be seen in Fig 4a–c, that the topological charge of each sub-lattice ($Q_{\text{A}}$, $Q_{\text{B}}$) reach an integral value of $\pm 1, \pm 2$ and $\pm 3$ for a threshold pulse width of $\tau _w =$0.7 ps, 3.1 ps and 12.8 ps, respectively, which indicates the formation of 1, 2 and 3 AFM skyrmions for the respective cases. To further confirm this, we also show the snapshots of magnetization corresponding to each case in Fig. 4d–f. These snapshots are taken at $t=200$ ps (measured from the beginning of the current injection). The total nucleation time ($\tau _{n}^{total}$) corresponding to each pulse width ($\tau _w$) is calculated from Fig. 4 and is listed in Table 1. For $\tau _w=0.7$ ps, a single AFM skyrmion is nucleated in time $\tau _{n}^{total} = 6.8$ ps. For $\tau _w=3.1$ ps, we obtain two AFM skyrmions in a total time of $\tau _{n}^{total} = 8.4$ ps. For $\tau _w=12.8$ ps where three AFM skyrmions are nucleated, the total nucleation time is $\tau _{n}^{total} = 16.8$ ps.

**Table 1:** Summary of Fig. 4: dependence of total nucleation time on current pulse width for multiple AFM skyrmions.

Pulse Width, $\tau _{w}$ (ps)	Topological charge, Q$_{A,B}$	AFM skyrmion count (n)	Total nucleation time, $\tau _{n}^{total}$ (ps)
0.7	$\pm 1$	One	6.8
3.1	$\pm 2$	Two	8.4
12.8	$\pm 3$	Three	16.8

The increase of total nucleation time $\tau _{n}^{total}$ for the case of two skyrmions is because of the following reasons. Initially, when the first AFM skyrmion is created, it is situated at the center of the film right below the contact region from which the current is being injected. The nucleation time for this skyrmion is dependent on the current density as discussed in Fig. 3. Moreover, for a fixed current amplitude, the nucleation time decreases with an increase in pulse width (for $\tau _w>\tau _w^0$). This is simply because the torque is applied for a longer duration allowing the magnetization under the contact region to reverse faster. This can be easily seen in Fig. 4b, where the time at which $Q_{\text{A,B}}$ reaches $\pm 1$ is $\sim 3$ ps. This is the nucleation time for the first skyrmion which is nearly half of the nucleation time of skyrmion in Fig. 4a. Once the first skyrmion is nucleated, for the creation of the second AFM skyrmion, the first AFM skyrmion has to be pushed outside the current injection region. In case of in-plane spin polarized current, this can happen without any external help if the current pulse is not switched off. This is due to the fact that the net torque due to spin current $J_{x,z}$ forces the nucleated AFM skyrmion to move towards $-y$-direction, leaving the current injection region free for subsequent AFM skyrmion nucleation. It is important to note that this is not possible when current is polarized out-of-plane ($J_{z,z}$) as there is no net torque on the AFM skyrmion in the in-plane direction. Hence, the process of nucleation of multiple skyrmion is exclusive to the application of in-plane polarized current ($J_{x,z}$). Due to this additional process of pushing of the first AFM skyrmion out of the injection region, the nucleation of second skyrmion takes longer than the first skyrmion. It may also be noted that during the time between $Q_{\text{A,B}}=\pm 1$ and $Q_{\text{A,B}}=\pm 2$, the second AFM skyrmion is already partially created but has a trivial topology similar to droplets described in Ref. ^59^. For Fig. 4b, the second skyrmion is nucleated at $t=8.4$ ps.

In Fig. 4c, we show the nucleation of three skyrmions for a pulse width of $\tau _w=12.8$ ps. The nucleation of first skyrmion in this case is similar to Fig. 4b and the first skyrmion is nucleated in 3 ps. During the nucleation of second skyrmion, as the current is being continuously injected, we observe an additional behavior during the nucleation process for which *Q* shows random switching between $Q=\pm 1$, and $Q=\pm 2$ [Fig. 4c], which further increases the t$^{total}_n$. As shown in the Supplementary Movie 4, this behavior corresponds to the merging of second AFM skyrmion with the first AFM skyrmion. As the current is injected in CPP geometry, STT decays outside the current injection region. As a result, even if the current pulse is not switched off, the first AFM skyrmion can only move a limited distance out of the current injection region. When the second AFM skyrmion is nucleated and pushed out of the current injection region, it merges with the slowly moving first skyrmion. However, when the current is switched off at 12.8 ps, then the second AFM skyrmion slows down, and it stabilizes without merging with the first AFM skyrmion while simultaneously allowing the formation of a third AFM skyrmion below the current injection area. This process of AFM skyrmion destruction did not happen during the creation of second skyrmion in Fig. 4b as the current pulse was already switched-off at 3.1 ps where $Q_{\text{A,B}}$ just reaches $\pm 1$. Similar to Fig. 4b, during the stage between $Q_{\text{A,B}}=\pm 2$ and $Q_{\text{A,B}}=\pm 3$, the third AFM skyrmion is already partially formed but each sub-lattice of this AFM skyrmion has a trivial topology ($Q_{\text{A,B}}=0$). The above nucleation process is also accompanied by a change in size and shape of the AFM skyrmions, which make the mechanism complex.

Finally, we note that despite the presence of complex features in the nucleation of multiple skyrmions leading to a non-monotonous variation in average nucleation time per skyrmion in each case, the phenomena itself is deterministic in nature and we can control the number of the nucleated AFM skyrmion by varying the pulse width of in-plane spin polarized current. Moreover, the direction of skyrmion propulsion is also dependent on the direction of spin polarization. Simply reversing the polarity of current in our simulations would lead to skyrmions being pushed towards $+y$-direction. In general, if the polarization direction of spin-current can be freely varied (in the plane), skyrmions can be forced to move in any arbitrary direction. This feature would be quite useful in logic-in-memory devices to create skyrmions on-demand and propel them towards different logic gates. It should, however, be noted that the forces on the AFM skyrmion weaken as the distance between AFM skyrmion and the current injection center increases. Therefore, to move skyrmions for longer distances, additional forces (for e.g., spin-orbit torque) would still be required.

**Figure 5:**
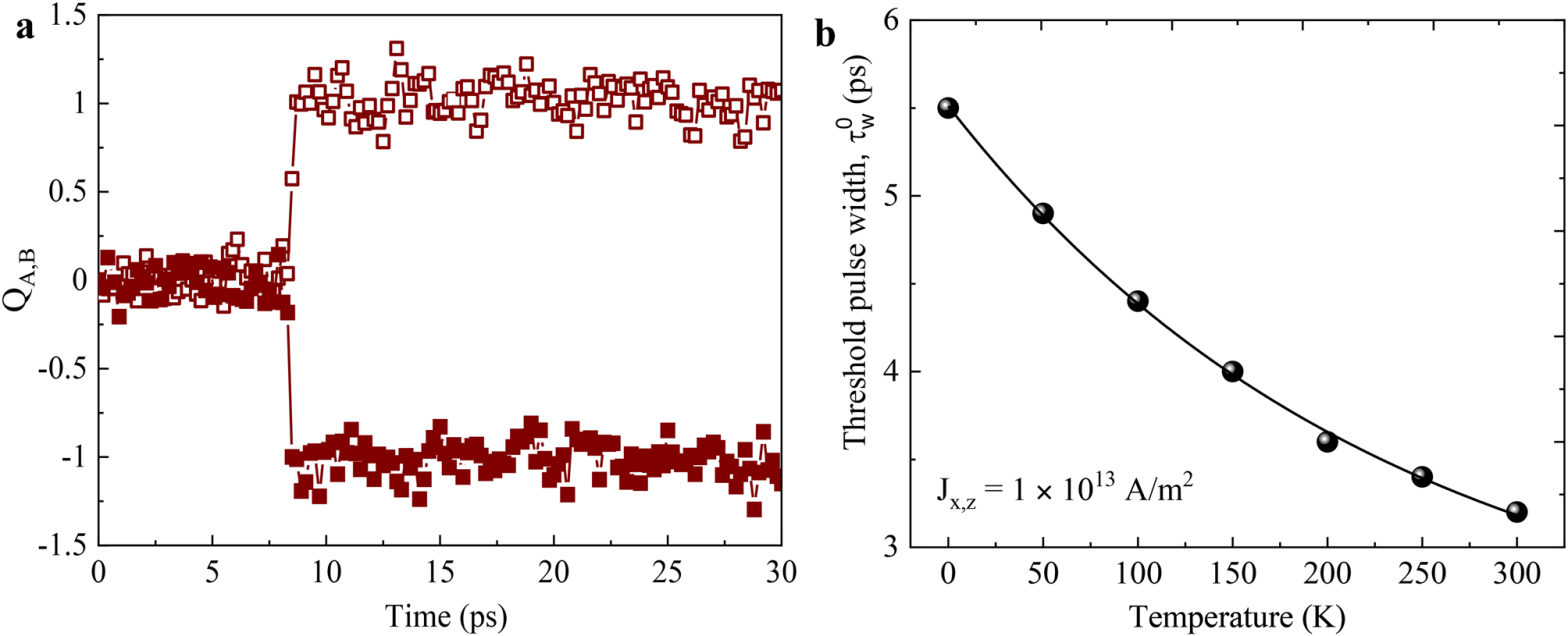
(**a**) Time evolution of topological charge at ($T =$300 K) for sub-lattice A (open symbols) and B (closed symbols) of the AFM skyrmion. (**b**) Variation of threshold pulse width on the temperature at fixed current density ($J_{x,z}=1 \times 10^{13}$ A/m$^2$).

### Temperature dependence

Finally, in order to check the robustness of our nucleation method using in-plane spin polarized current, we introduce thermal noise in the system. This thermal noise is added as a fluctuating magnetic field in accordance to the formulations given by Brown^60^. Figure 5a shows the topological charge of sub-lattice A and B during the nucleation at $T =$300 K, which is obtained for pulse width $\tau _w =$5.5 ps and current density $J_{{x},{z}}=1.0 \times 10^{13}~\rm {A/m}^2$. Despite the additional noise due to the finite temperature, the nucleation time can still be accurately extracted since the abrupt change in $Q_{\text{A,B}}$ due to the nucleation of AFM skyrmion dominates over the noise in the data of $Q_{\text{A,B}}$ due to the finite temperature. For the case shown in Fig. 5a, the nucleation time is found to be $\sim 8.5$ ps which is a bit lower compared to the nucleation time at $T =$ 0 K in Fig. 1b. However, for a fixed $\tau _w=5.5$ ps and $J_{{x},{z}}=1.0 \times 10^{13}~\rm {A/m}^2$, the nucleation time $\tau _n$ is found to be nearly constant (variation $\le 2$ ps) as a function of temperature. In order to determine nucleation time accurately, we average simulations with ten different random seeds for each temperature. In Fig. 5b, we show the dependence of threshold pulse width $\tau _w^0$ on temperature at fixed $J_{{x},{z}}=1.0 \times 10^{13}~\rm {A/m}^2$ for the nucleation of a single skyrmion. $\tau _w^0$ is found to decrease by $\sim 45\%$ as temperature increases from 0 K to 300 K. The decrease in $\tau _w^0$ is because of the additional thermal noise which helps in destabilizing the initial checkerboard AFM configuration allowing for a faster reversal when the current is applied. Physically, the inclusion of thermal noise can be seen as an additional contribution to the initial energy of the system (checkerboard AFM state) making it easier for the system to cross the energy barrier [in Fig. 2a] between the checkerboard AFM state and AFM skyrmion state leading to lower energy expenditure (less current) for AFM skyrmion nucleation at higher temperatures. These results show that our method of nucleation of AFM skyrmions is robust and finite temperature improves overall nucleation phenomenon by reducing threshold pulse width.

## Conclusion

We investigate the nucleation of AFM skyrmions by in-plane spin polarized current. We find that utilizing in-plane polarization for skyrmion nucleation offers more than one order of magnitude lower energy consumption compared to the traditionally used out-of-plane polarized current. Moreover, for same values of injected current, the time required for nucleation of skyrmion is significantly lower for in-plane polarized current. From our simulations, we find a nucleation time between 12–7 ps for injected current density in the range of $1-3\times 10^{13}~\rm {A/m}^2$. Threshold current for nucleation in the in-plane case is nearly one order lower than the out-of-plane case, allowing for the creation of skyrmion at much lower currents. We also show the possibility of nucleating multiple skyrmions by injecting current pulses longer than the required threshold pulse width, a phenomenon which is exclusive to the use of in-plane polarized current. Finally, we show that our nucleation method is robust against temperature effects and the threshold pulse width decreases with the additional thermal noise in the system, which is advantageous in terms of energy consumption.

## Methods

We solve Eq. (1) using the micromagnetic simulation package Mumax$^3$ [Ref. ^55^]. Material parameters are taken for Refs.^54,56^, we use the exchange stiffness $A_{ex}=-10$ pJ/m, Gilbert damping coefficient $\alpha = 0.3$, the saturation magnetization $M_{\text{s}} = 580 \times 10{^3}$ A/m , the DMI constant $D = 3.5 \times 10^{-3}$ J/m$^2$, and the PMA constant $K = 0.8 \times 10^6$ J/m$^3$. These parameters correspond to $\epsilon =\frac{D}{2\sqrt{AK_{\text{eff}}}}=0.7213$ and $\kappa =\frac{2K_{\text{eff}}}{\mu _0M_S^2}=2.7849$ which are dimensionless DMI and anisotropy constant, respectively. Here, $K_{\text{eff}}$ is the effective anistropy strength after overcoming the magnetostatic interactions ($K_{\text{eff}}=K-\mu _0M_S^2/2$). The polarization efficiency of the spin-polarized current is $|P|= 0.56$. The field-like torque is assumed to be zero ($\xi =0$). Since we utilize CPP (current perpendicular to plane) geometry the spin current is always flowing in the *z*-direction along the thickness of the film ($J=J_0{\hat{z}}$). We assume that the magnetization does not change along the thickness of the film, for which the non-adiabatic torque acting on the system vanishes and is not considered in our simulations. As Mumax$^3$ solves Eq. (1) on finite difference grid, for small enough cell sizes, it is equivalent to an atomistic model and is sufficiently valid for an AFM system^47,54,56^. In our simulations, we have considered an AFM square thin film of dimensions $512~\rm nm\times 512~\rm nm \times 2~\rm nm$ which is discretized into cuboidal cells of size $1~\rm nm\times 1~\rm nm \times 2~\rm nm$. The dimensions of each cell are kept much smaller than the exchange length $l_{ex}=\sqrt{A/K}=3.53$ nm. We employ periodic boundary conditions along both *x* and *y* direction to avoid any interaction of the AFM skyrmion with the edges. For finite temperature simulations, the additional random thermal field^60^ is given by $\mathbf {B_{therm}} = \mathbf {\eta }\sqrt{\frac{2 \alpha k_B T}{M_{S}\gamma \Delta V \Delta t}}$ where, $\mathbf {\eta }$ represents a random vector drawn from a normal distribution, $\Delta V$ is the micromagnetic cell volume, $\gamma$ is the gyromagnetic ratio of the electron, $\Delta t$ is the time-step for integrating Eq. (1) and *T* represents the temperature.

## Supplementary information

Supplementary Legends.

Supplementary Video 1.

Supplementary Video 2.

Supplementary Video 3.

Supplementary Video 4.
