# Supplementary material for: Energy-efficient ultrafast nucleation of single and multiple antiferromagnetic skyrmions using in-plane spin polarized current
Source: Sci Rep. 2021 Jun 10;11:12332. doi: 10.1038/s41598-021-91591-8 (PMC8192941; doi:10.1038/s41598-021-91591-8)
Supplement: Supplementary file 1 — Supplementary Legends. [file 41598_2021_91591_MOESM1_ESM.pdf]

Supplementary Material : Energy-efficient ultrafast nucleation of single and multiple antiferromagnetic skyrmions using in-plane spin polarized current

Kacho Imtiyaz Ali Khan[1], **Naveen Sisodia**[1], **and P. K. Muduli**[1,*]

[1]Department of Physics, Indian Institute of Technology Delhi, Hauz Khas, New Delhi-110016, India
[*]muduli@physics.iitd.ac.in

Supplementary Movie 1. Nucleation of AFM skyrmion using in-plane spin polarized current ($J_{x,z} = 1 \times 10^{13}\,\mathrm{A/m^2}$) with a pulse width of $\tau_w = 5.5$ ps injected at the centre of the AFM thin film (200 nm x 200 nm) within a circular area (diameter 70 nm). The colorbar represents the magnitude of the $z-$ component of the magnetization, m_z.

Supplementary Movie 2. Time evolution of individual magnetic moments of sub-lattice A and B as an in-plane spin current ($J_{x,z} = 6.5 \times 10^{13}\,\mathrm{A/m^2}$) polarized along x-direction is injected. The evolution is shown until the magnetization under the injection region reaches the in-plane configuration. The white color represents $m_z = +1$ (sub-lattice-A), the black color represents $m_z = -1$ (sub-lattice-B), and the red color represents $m_z = 0$ (in-plane configuration of both sub-lattice A and B).

Supplementary Movie 3. Time evolution of individual magnetic moments of sub-lattice A and B as an out-of-plane spin polarized current ($J_{z,z} = 6.5 \times 10^{13}\,\mathrm{A/m^2}$) polarized along z-direction is injected. The evolution is shown until the magnetization under the injection region reaches the in-plane configuration. The white color represents $m_z = +1$ (sub-lattice-A), the black color represents $m_z = -1$ (sub-lattice-B), and the red color represents $m_z = 0$ (in-plane configuration of both sub-lattice A and B).

Supplementary Movie 4. Nucleation of three AFM skyrmions using in-plane spin polarized current ($J_{x,z} = 3 \times 10^{13}\,\mathrm{A/m^2}$) with a pulse width equal to the thershold pulse width ($\tau_w^0 = 0.7$ ps) injected at the centre of the AFM thin film (200 nm x 200 nm) within a circular area (diameter 70 nm). The colorbar represents the magnitude of the $z-$ component of the magnetization, m_z.
